# Alpha-1-Antitrypsin Deficiency and Bronchiectasis: A Concomitance or a Real Association?

**DOI:** 10.3390/ijerph17072294

**Published:** 2020-03-29

**Authors:** Alessandro Sanduzzi, Emanuele Ciasullo, Ludovica Capitelli, Stefano Sanduzzi Zamparelli, Marialuisa Bocchino

**Affiliations:** 1 Department of Clinical Medicine and Surgery, Section of Respiratory Disease, University Federico II, AORN dei Colli-Monaldi Hospital, 80138 Naples, Italy; ema.ciasullo@gmail.com (E.C.); ludovica.capitelli@gmail.com (L.C.); marialuisa.bocchino@unina.it (M.B.); 2 University Luigi Vanvitelli, U.O.C. Clinica Pneumologica SUN, AORN dei Colli-Monaldi Hospital, 80138 Naples, Italy; stefanosanduzzi@gmail.com

**Keywords:** alpha-1-antitrypsin deficiency, emphysema, bronchiectasis, respiratory infections

## Abstract

Alpha-1-antitrypsin deficiency (AATd) is a hereditary disease, mainly characterized by early onset and the lower lobes’ predominant emphysema. Bronchiectasis is characterized by dilatation of the bronchial wall and a clinical syndrome whose features are a cough, sputum production and frequent respiratory exacerbations. In the literature, there are many papers concerning these two clinical entities, but there is still a lot of debate about a possible association between them, in particular about the frequency of their association and causal links. The aim of this short communication is to show the literature reports about the association between AATd and bronchiectasis to establish the state of the art and possible future developments in this research field.

## 1. Introduction

Alpha-1-antitrypsin deficiency (AATd) is a rare and often underdiagnosed hereditary disease, showing a prevalence of 1–5 cases out of 10,000. AAT is the prototype of the endogenous protease inhibitor (Pi) of serine proteases; it is coded by the serine-protease inhibitor (SERPINA1) gene and is secreted in the blood mainly by the liver. There are several genetic variants (generally represented by point mutations in the gene sequence leading to aminoacid substitutions) which may alter the electrophoretic mobility of the resulting protein. According to their electrophoretic mobility, these variants are labelled A–Z, i.e., faster or slower, compared to the most common (normal variant), labelled M. Pathological variants are defined: deficient variants (point mutation that causes retention of the AAT in the epathocite cytoplasma and poor blood secretion), null mutations (generally caused by the presence of a stop codon resulting in the absence of AAT in serum) or dysfunctional variants (point mutation that lead to the abnormal function of AAT with reduced activity). Common pathological variants are S and Z, with MM, MS, MZ, SS, SZ and ZZ protein phenotypes accounting for the majority of genotypes.

AATd is associated with an increased risk of chronic obstructive pulmonary disease (COPD), in particular early onset and lower lobes’ predominant panlobular emphysema, which is often out of proportion considering patient’s smoking history, but in some cases airways obstruction is not present. Symptoms and signs include a chronic cough, sputum, and progressive dyspnea on exertion until chronic respiratory failure. The diffusing capacity of carbon monoxide (DLCO) is more sensitive than FEV_1_ as an indicator for the presence, progression and severity of emphysema. The involvement of the peripheral airways in AATd is less well described, but patients also may present asthma. Other extra-thoracic manifestations in AATd are liver disease (with a possible evolution towards cirrhosis), granulomatosis with polyangiitis, and panniculitis [1]. Diagnosis is performed by the determination of the serum levels of AAT, protein phenotyping by electrophoresis, and genotyping where specific primers for known mutations are available; whole-exon sequencing may be required if there are no primers available or null variants are expected (intron sequencing is currently uncertain).

On the other hand, bronchiectasis is a clinical syndrome characterized by a chronic cough, mucopurulent sputum production and recurrent respiratory infections, with abnormal and permanent bronchial dilatation in high-resolution computed tomography (HRCT). These findings may result from a number of possible causes, and these may influence treatment and prognosis. It is not a rare disease, but it is rarely diagnosed, or diagnosed late, since the only way to identify such an entity is HRCT, a tool not so frequently used by clinicians.

A controversial issue is the association between AATd and bronchiectasis: in fact, the literature shows opposite reports. Thrrefore, our aim is to investigate possible links between these two clinical entities, in order to highlight any cause/effect relationship, helping clinicians in managing patients with AATD and/or bronchiectasis.

## 2. Materials and Methods

A literature search was conducted by using the keywords (alpha-1-antitrypsin deficiency, emphysema, bronchiectasis, respiratory infections) as search terms and probing for them in PubMed and reviewing European Respiratory Journal Supplements. Articles, case reports, guidelines, and reviews were screened. Whenever there was an overlap of information, the more recent papers were selected for inclusion.

## 3. Findings

The association between AATd and bronchiectasis is controversial and there is a lot of debate about this issue.

The first point to consider is the incidence of AATd among patients affected by bronchiectasis, and a possible pathogenetic role of AATd in bronchiectasis etiology. Chan et al. underline that there is a higher frequency of patients suffering from AATd showing bronchiectasis [2]. In fact, AAT is present in lung tissue, with extracellular lung fluid levels of about 10% serum concentration [3] and is involved in the regulation of different inflammatory pathways, so that AAT deficiency would promote bronchial wall inflammation and damage, leading to bronchiectasis. In particular, AAT is the prototype of the endogenous inhibitor of serine proteases such as neutrophil elastase (NE), which is responsible for direct damage to lung epithelial cells and supportive tissues. Moreover, it has been shown that NE could help bacterial survival because its proteolytic action could alter some extracellular soluble anti-pathogen substances such as immunoglobulins, complement components, defensins, lysozyme, lactoferrin, and cathelicidins [4,5]. Therefore, antagonizing NE proteolytic activity, AAT protects the function of such soluble anti-pathogen substances and promotes clearance of extracellular bacteria preserving cilia-mediated bacterial clearance. Another NE activity interfering with bacterial clearance is the increased expression of a cell-surface mucine (MUC1) which is a bacterial binding site, promoting bacterial invasion into epithelial cells: this internalization shields bacteria from the extracellular immune effectors cited above. Therefore, AAT anti-NE effect may promote bacterial killing by prolonging exposure of bacteria to such soluble extracellular anti-microbial mediators and phagocytic cells [6]. Moreover, Petrache et al. [7] have shown that AAT directly inhibits the activity of caspase-3, an intracellular cysteine protease involved in neutrophil apoptosis. AAT is also probably involved in the inhibition of gelatinase B (MMP-9) in neutrophils (a molecule that contributes to neutrophil extravasation and stem cell mobilization via the degradation of basement membrane collagens) [8].

In addition, some recent studies support the hypothesis that AAT seems to have immune-modulatory functions other than its classic anti-neutrophil protease activity [9]. In particular, Jonigk et al. [10] demonstrated that exogenous AAT, independent of its anti-elastase activity, can significantly lower the lipopolysaccharide (LPS)-induced release of two molecules involved in different inflammatory pathways: Tumor Necrosis Factor-α (TNF-α) and Interleukin-8 (IL-8). Bergin et al. [11] and O’Dwyer et al. [12] showed that AAT can directly bind IL-8 and leukotriene-B4 (LTB4), inhibiting their chemoattractant activities. In addition, AAT downregulates TNF-α gene expression by inhibiting the nuclear factor kappa-light-chain-enhancer of activated B cells (NF-κB) signaling [13] and reduces LPS-induced synthesis and the release of active IL-1β [14], which is an important inflammatory mediator.

All these studies are supported by another demonstration of AAT’s anti-inflammatory function: in fact, Zhu et al. [15] showed that AAT has a protective effect on the development of Ventilator-Induced Lung Injury (VILI): in rats treated with high tidal volume ventilation, the bronchoalveolar lavage fluid (BALF) levels of proinflammatory cytokines (TNF-α, IL-1β, and IL-6) were markedly reduced when rats received intravenous administration of AAT, whereas the level of anti-inflammatory IL-10 was significantly increased. Therefore, AAT can ameliorate VILI by inhibiting inflammatory mediator production and authors even suggest that the administration of AAT may represent an interventional approach for ARDS patients who need mechanical ventilation.

The hypothesis of a protective role of AAT on bronchial inflammation is supported by two other studies, one in mice and the other in humans. The first one shows that in AAT^+/+^ transgenic mice that express human AAT in lungs, the mortality of Pseudomonas aeruginosa-induced pneumonia was reduced of 90% compared to non-transgenic control animals. Moreover, exogenous human AAT given to non-transgenic mice also significantly reduced P. aeruginosa pneumonia mortality. P. aeruginosa-infected AAT^+/+^ mice demonstrated reduced lung tissue damage, decreased bacterial concentrations in the lungs and blood, and diminished circulating cytokine concentrations compared to infected non-transgenic mice [16]. The second study, instead, shows that among subjects with humoral immunodeficiencies requiring gamma–globulin replacement therapy, patients with bronchiectasis were found to have lower median levels of AAT than those without bronchiectasis [17], suggesting that a deficiency of AAT could promote the development of bronchiectasis.

Another interesting finding is reported in a recent analysis of the US Bronchiectasis Research Registry by Eden et al. [18] about the frequency of Non-Tubercolous Mycobacteria (NTM) infection in patients with bronchiectasis and AATd. In fact, NTM infections are known to be common in patients with bronchiectasis, but, surprisingly, in AATd patients with bronchiectasis there was a greater percentage of patients reporting NTM on sputum culture compared with idiopathic bronchiectasis; 62.8% of patients with AATd versus 44.9% of patients without AATd. These findings are supported by the evidence that AAT inhibits rapidly growing mycobacterial infection in macrophages [19].

Evidence of how AAT deficiency promotes inflammation is reported above, but another interesting hypothesis to explain the pathogenetic role of AATd in the development of bronchiectasis concerns the pro-inflammatory of mutated AAT isoforms secreted by the liver in blood and stored in lung tissue. In fact, the Z isoform of AAT could polymerize in the lung, acting as a chemotactic factor for neutrophils, with NE release leading to damage of the bronchial wall, and finally to bronchiectasis. According to this hypotesis, Araujo et al. showed an intriguing association between phenotype ZZ and the severity of bronchiectasis, independent of the presence of emphysema: within 110 patients with AATd, 26 were found to have bronchiectasis; in this group, mean value of AAT was 59.9 mg/dL; the ZZ phenotype was the most prevalent (42.3%); and there was a correlation between the severity of AATd and bronchiectasis, with a preponderance of the phenotype ZZ (100%) within the group with the most severe cases. In particular, ZZ patients had a higher rate of exacerbations, hospital admissions, and frequency and persistence of colonization by P. aeruginosa [20]. Non-Z isoforms likely also play a pro-inflammatory role: in fact, Dandurand et al. conducted a case-controlled study about bronchiectasis prevalence, comparing 16 COPD subjects with non-PiZ phenotypes (3 SS, 8 MS, 5 MZ) with 16 COPD patients matched for age, sex and FEV_1_ with AAT levels ≤ 1.15 g/L, but MM genotype (MM), and with 16 similarly matched COPD patients with serum AAT levels ≥ 1.15 g/L and not genotyped. Bronchiectasis was more frequent in non-PiZ phenotype AATD subjects than in MM genotype subjects and COPD subjects with normal serum AAT levels [21].

Moreover, two similar case reports describe patients with non-PiZ phenotypes AATd with moderate low levels of AAT, whose HRCT showed, suprisingly, only the presence of bronchiectasis but no emphysema. In both cases, other causes of bronchiectasis were excluded. The first one is a case of a 52-year-old man, a native of Naples (Italy), nonsmoker, admitted for persistent cough accompanied by low-grade fever and dyspnea. His HRCT showed upper right lobe and medium lobe bronchiectasis but no emphysema. AAT level was 85 mg/dL. The gene sequencing revealed the presence of a new mutation in the heterozygous state, probably responsible for the S bandage obtained by electrophoresis; this mutation corresponded with a new S allelic variant, not identified before, that the authors called S-Napoli [22]. The second one is the case of a 52-year-old woman nonsmoker, with an history of frequent pulmonary exacerbations, with bilateral bronchiectasis at the middle lobe and the left lower lobe with no emphysema. AAT level was 78 mg/dL. The gene sequencing showed the presence of a novel missense mutation in a heterozygous state on a M3 allele [23].

Conversely, Shin et al. [24] found that the frequency of the association between AATd and bronchiectasis is not relevant. Lonni et al. [25], in a population of 1258 bronchiectasis patients enrolled from different European countries, found only eight cases of AATd (0,6%). Pasteur et al. [26] reported that there were neither patients with PiZZ phenotype, nor a significant increase in the frequency of partial deficiency AAT genotypes (PiMZ, PiMS) in patients with bronchiectasis. Different phenotypes showed the same frequency in such a group and in overall population in the United Kingdom; all the bronchiectasis patients had a normal serum level of AAT (between 0.9 and 1.8 g/L), while in only two Pi-MZ subjects was there a lower value (0.7 and 0.85, respectively).

On the other hand, an interesting question is: how many patients suffering from AATd develop bronchiectasis? Cortese et al., on behalf of Italian Registry of AATd, underline that 58 cases of bronchiectasis (12%) have been identified in a cohort of 475 patients suffering from AATd, but prevalence is clearly higher if we consider only subjects who performed HRCT (58 out of 193, i.e., 30%) [27]. Similarly, Parr and coworkers describe a prevalence of 27% of bronchiectasis in subjects with PiZ genothype (20 out of 74) [28]. Moreover, a similar result is also reported by a retrospective study of 114 AATD patients in a Portuguese hospital, who underwent a CT scan: 29.8% had HRCT evidence of bronchiectasis, AAT mean serum level was 66.0 ± 33.9 mg/dL, and patients with bronchiectasis had lower AAT serum levels (*p* = 0.015). The presence of bronchiectasis was related to lower AAT serum levels and to the presence of emphysema, although >1/3 of patients had bronchiectasis without radiological evidence of emphysema [29].

## 4. Conclusions

Our research displays an increasing number of papers about AATd and bronchiectasis, but the association is still controversial, even if, as reported above, there is a lot of evidence that could suggest a pathogenetic role of AATd in bronchiectasis development. Therefore, the questions are:

(1) Is it necessary to screen for bronchiectasis development in patients with AATd?;

(2) Is it necessary to screen for AATd as a cause in patients with bronchiectasis?

Regarding the first point, the European Respiratory Society statement for AATd include bronchiectasis among underlying, possible, comorbidities in AAT deficiency but there is not a clear recommendation to screen bronchiectasis in AATd [30].

About the second question, European Respiratory Society Guidelines for the management of adult bronchiectasis recommend only three aetiological tests in a patient with a new diagnosis of bronchiectasis: differential blood count, serum immunoglobulins and tests for allergic bronchopulmonary aspergillosis. The expert panel suggests that only the presence of lower lobes emphysema or early onset airways’ obstruction could represent an indication to screen for AATd [31]. Similarly, British Thoracic Society guidelines for bronchiectasis in adults state that there is insufficient information to decide which bronchiectasis patients must been screened for AATd, therefore, in this case authors also limit the screening to cases of basal panlobular emphysema [32]. Considering this limited AATd screening in bronchiectasis patients, although the data presented above document a possible role of AATd as an underlying etiology of bronchiectasis, the majority of bronchiectasis patients likely never received AATd screening. In fact, in 2014 the Italian Respiratory Society (IRS/SIP) conducted a national audit on adult patients with bronchiectasis attending secondary care hospitals in Italy; only 8.2% were tested for AATd [33].

We believe that, in patients with bronchiectasis, a preliminary low-cost screening of AATd (dosing serum AAT together with C Reactive Protein) could be useful, because it could considerably alter the clinical management of patients, offering the possibility of an eventual adjunctive-specific therapeutic intervention (AAT replacement therapy).

In conclusion, such an issue deserves higher regard in the management of both diseases, but mainly in bronchiectasis patients, where possible therapeutic strategies of AATd could improve the management of such disease.

## References

[1] Cazzola M., Stolz D., Rogliani P. (2020). α1-Antitrypsin deficiency and chronic respiratory disorders. Eur. Respir. Rev..

[2] Chan EDWooten W.I., Hsieh E.W.Y., Johnston K.I., Shaffer M., Dandhaus R., van de Veerdonk F. (2019). Diagnostic evaluation of bronchiectasis. Respir. Med. X.

[3] Wewers M.D., Casolaro M.A., Sellers S.E., Swayze S.C., McPhaul K.M., Wittes J.T., Crystal R.G. (1987). Replacement therapy for alpha 1-antitrypsin deficiency associated with emphysema. N. Engl. J. Med..

[4] Moraes T.J., Chow C.W., Downey G.P. (2003). Proteases and lung injury. Crit. Care Med..

[5] Stockley R.A. (1995). Role of inflammation in respiratory tract infections. Am. J. Med..

[6] Parker D., Prince A. (2011). Innate immunity in the respiratory epithelium. Am. J. Respir. Cell Mol. Biol..

[7] Petrache I., Fijalkowska I., Medler T.R., Skirball J., Cruz P., Zhen L., Horia I.P., Terence R., Flotte R., Tuder M. (2006). Alpha-1 antitrypsin inhibits caspase-3 activity, preventing lung endothelial cell apoptosis. Am. J. Pathol..

[8] Opdenakker G., Fibbe W.E., Van Damme J. (1998). The molecular basis of leukocytosis. Immunol. Today.

[9] Janciauskiene S., Wrenger S., Immenschuh S., Olejnicka B., Greulich T., Welte T., Chorostowska-Wynimko J. (2018). The Multifaceted Effects of Alpha1-Antitrypsin on Neutrophil Functions. Front Pharmacol..

[10] Jonigk D., Al-Omari M., Maegel L., Müller M., Izykowski N., Hong J., Hong K., Kim S.H., Dorsch M., Mahadeva R. (2013). Anti-inflammatory and immunomodulatory properties of α1-antitrypsin without inhibition of elastase. Proc. Natl. Acad. Sci. USA.

[11] Bergin D.A., Reeves E.P., Meleady P., Henry M., McElvaney O.J., Carroll T.P., Condron C., Chotirmall S.H., Clynes M., O’Neill S.J. (2010). α-1 Antitrypsin regulates human neutrophil chemotaxis induced by soluble immune complexes and IL-8. J. Clin. Investig..

[12] O’Dwyer C.A., O’Brien M.E., Wormald M.R., White M.M., Banville N., Hurley K., McCarthy C., McElvaney N.G., Reeves E.P. (2015). The BLT1 Inhibitory Function of α-1 Antitrypsin Augmentation Therapy Disrupts Leukotriene B4 Neutrophil Signaling. J. Immunol..

[13] Bergin D.A., Reeves E.P., Hurley K., Wolfe R., Jameel R., Fitzgerald S., McElvaney N. (2014). The circulating proteinase inhibitor α-1 antitrypsin regulates neutrophil degranulation and autoimmunity. Sci. Transl. Med..

[14] Aggarwal N., Korenbaum E., Mahadeva R., Immenschuh S., Grau V., Dinarello C.A., Welte T., Janciauskiene S. (2016). α-Linoleic acid enhances the capacity of α-1 antitrypsin to inhibit lipopolysaccharide induced IL-1β in human blood neutrophils. Mol. Med..

[15] Zhu H., He J., Liu J., Zhang X., Yang F., Liu P., Wang S. (2018). Alpha 1-antitrypsin ameliorates ventilator-induced lung injury in rats by inhibiting inflammatory responses and apoptosis. Exp. Biol. Med. (Maywood).

[16] Pott G.B., Beard K.S., Bryan C.L., Merrick D.T., Shapiro L. (2013). Alpha-1 Antitrypsin Reduces Severity of *Pseudomonas* Pneumonia in Mice and Inhibits Epithelial Barrier Disruption and *Pseudomonas* Invasion of Respiratory Epithelial Cells. Front. Public Health.

[17] Peppers B.P., Zacharias J., Michaud C.R., Frith J.A., Varma P., Henning M., Quinn L.M., Tcheurekdjian H., Craig T., Hostoffer R.W. (2018). Association between α1-antitrypsin and bronchiectasis in patients with humoral immunodeficiency receiving gammaglobulin infusions. Ann. Allergy Asthma Immunol..

[18] Eden E., Choate R., Barker A., Addrizzo-Harris D., Aksamit T.R., Daley C.L., Daniels M.L.A., DiMango A., Fennelly K., Griffith D.E. (2019). The clinical features of bronchiectasis associated with alpha-1 antitrypsin deficiency, common variable immunodeficiency and primary ciliary dyskinesia—results from the U.S. Bronchiectasis Research Registry. Chronic Obstr. Pulm. Dis..

[19] Chan E.D., Kaminska A.M., Gill W., Chmura K., Feldman N.E., Bai X., Floyd C.M., Fulton K.E., Huitt G.A., Strand M.J. (2007). Alpha-1-antitrypsin (AAT) anomalies are associated with lung disease due to rapidly growing mycobacteria and AAT inhibits Mycobacterium abscessus infection of macrophages. Scand. J. Infect. Dis..

[20] Araujo D., Sucena M. (2015). Association between alpha 1 antitrypsin and bronchiectasis. Eur. Respir. J..

[21] Dandurand R., Carroll T., Estépar R.S.J., Bourbeau J., Eidelman D. (2014). Bronchiectasis but not emphysema is more prevalent in non-PiZ alpha-1 antitrypsin deficiency (AATD) COPD than in usual COPD. Eur. Respir. J..

[22] Mosella M., Accardo M., Molino A., Maniscalco M., Zamparelli A.S. (2018). Description of a new rare alpha-1 antitrypsin mutation in Naples (Italy), PI*M S-Napoli. Ann. Thorac. Med..

[23] Carpagnano G.E., Santacroce R., Palmiotti G.A., Leccese A., Giuffreda E., Margaglione M., Barbaro M.P.F., Aliberti S., Lacedonia D. (2017). A New SERPINA-1 Missense Mutation Associated with Alpha-1Antitrypsin Deficiency and Bronchiectasis. Lung.

[24] Shin M.S., Ho K.J. (1993). Bronchiectasis in patients with a1-antitrypsin deficiency: A rare occurrence?. Chest.

[25] Lonni S., Chalmer2 J.D., Goeminne P.C., McDonnell M.J., Dimakou K., de Soyza A., Polverino E., van de Kerkhove C., Rutherford R., Davison J. (2015). Etiology of Non–Cystic Fibrosis Bronchiectasis in Adults and Its Correlation to Disease Severity. AnnalsATS.

[26] PastEur M.C., Helliwell S.M., Houghton S.J., Webb S.C., Foweraker J.E., Coulden R.A., Flower C.D., Bilton D., Keogan M.T. (2000). An investigation into causative factors in patients with bronchiectasis. Am. J. Respir. Crit. Care Med..

[27] Cortese R., Mennitti C., Mariani F., Piloni D., Aliberti S., Ottaviani S., Ferrarotti I., Corsico A. (2016). Bronchiectasis in patients with alpha1-antitrypsin deficiency: Prevalence and characteristics. Eur. Respir. J..

[28] Parr D.G., Guest P.G., Reynolds J.H., Dowson L.J., Stockley R.A. (2007). Prevalence and impact of bronchiectasis in alpha1-antitrypsin deficiency. Am. J. Respir. Crit. Care Med..

[29] Costa A.F.C.E., Farinha I. (2019). Bronchiectasis in alpha1-antitrypsin deficiency. Eur. Respir. J..

[30] Miravitlles M., Dirksen A., Ferrarotti I., Koblizek V., Lange P., Mahadeva R., McElvaney N.G., Parr D., Piitulainen E., Roche N. (2017). European Respiratory Society statement: Diagnosis and treatment of pulmonary disease in α_1_-antitrypsin deficiency. Eur. Respir. J..

[31] Polverino E., Goeminne P.C., McDonnell M.J., Aliberti S., Marshall S.E., Loebinger M.R., Murris M., Cantón R., Torres A., Dimakou K. (2017). European Respiratory Society guidelines for the management of adult bronchiectasis. Eur. Respir. J..

[32] Hill A.T., Sullivan A.L., Chalmers J.D., de Soyza A., Elborn J.S., Floto R.A., Grillo L., Gruffydd-Jones K., Harvey A., Haworth C.S. (2018). British Thoracic Society guideline for bronchiectasis in adults. BMJ Open Resp. Res..

[33] Aliberti S., Hill A.T., Mantero M., Battaglia S., Centanni S., Cicero S.L., Lacedonia D., Saetta M., Chalmers J.D., Blasi F. (2016). SIP bronchiectasis audit working group. Quality standards for the management of bronchiectasis in Italy: A national audit. Eur. Respir. J..

